# Arg913Gln variation of SLC12A3 gene is associated with diabetic nephropathy in type 2 diabetes and Gitelman syndrome: a systematic review

**DOI:** 10.1186/s12882-019-1590-9

**Published:** 2019-10-28

**Authors:** Eduardo De la Cruz-Cano, Cristina del C. Jiménez-González, Vicente Morales-García, Conny Pineda-Pérez, Juan G. Tejas-Juárez, Francisco J. Rendón-Gandarilla, Silvia Jiménez-Morales, José A. Díaz-Gandarilla

**Affiliations:** 1 División Académica Multidisciplinaria de Comalcalco, Universidad Juárez Autónoma de Tabasco, C. P. 86650 Comalcalco, Tabasco Mexico; 2 Secretaría de Salud, Hospital General de Comalcalco, Departamento de Laboratorio de Análisis Clínicos, C.P. 86300 Comalcalco, Tabasco Mexico; 3 División Académica de Ciencias de la Salud, Universidad Juárez Autónoma de Tabasco, C.P. 86100 Villahermosa, Tabasco Mexico; 4 Instituto Nacional de Medicina Genómica (INMEGEN), Laboratorio Genómica del Cáncer, Periférico Sur No. 4809, Col. Arenal Tepepan, Deleg. Tlalpan, C.P. 14610 Ciudad de México, Mexico; 5 Universidades para el Bienestar Benito Juárez García, Medicina Integral y Salud Comunitaria, Juan R. Escudero, Guerrero, C.P. 39940 Mexico

**Keywords:** Diabetic nephropathy, Type 2 diabetes mellitus, Gitelman syndrome, SLC12A3 gene

## Abstract

**Abstract:**

**Background:**

Diabetic nephropathy is a global common cause of chronic kidney disease and end-stage renal disease. A lot of research has been conducted in biomedical sciences, which has enhanced understanding of the pathophysiology of diabetic nephropathy and has expanded the potential available therapies. An increasing number of evidence suggests that genetic alterations play a major role in development and progression of diabetic nephropathy. This systematic review was focused on searching an association between Arg913Gln variation in SLC12A3 gene with diabetic nephropathy in individuals with Type 2 Diabetes and Gitelman Syndrome.

**Methods:**

An extensive systematic review of the literature was completed using PubMed, EBSCO and Cochrane Library, from their inception to January 2018. The PRISMA guidelines were followed and the search strategy ensured that all possible studies were identified to compile the review. Inclusion criteria for this review were: *1)* Studies that analyzed the SLC12A3 gene in individuals with Type 2 Diabetes and Gitelman Syndrome. *2)* Use of at least one analysis investigating the association between the Arg913Gln variation of SLC12A3 gene with diabetic nephropathy. *3)* Use of a case–control or follow-up design. *4)* Investigation of type 2 diabetes mellitus in individuals with Gitelman’s syndrome, with a history of diabetic nephropathy.

**Results:**

The included studies comprised 2106 individuals with diabetic nephropathy. This review shows a significant genetic association in most studies in the Arg913Gln variation of SLC12A3 gene with the diabetic nephropathy, pointing out that the mutations of this gene could be a key predictor of end-stage renal disease.

**Conclusions:**

The results showed in this systematic review contribute to better understanding of the association between the Arg913Gln variation of SLC12A3 gene with the pathogenesis of diabetic nephropathy in individuals with T2DM and GS.

## Background

**Fig. 1:**
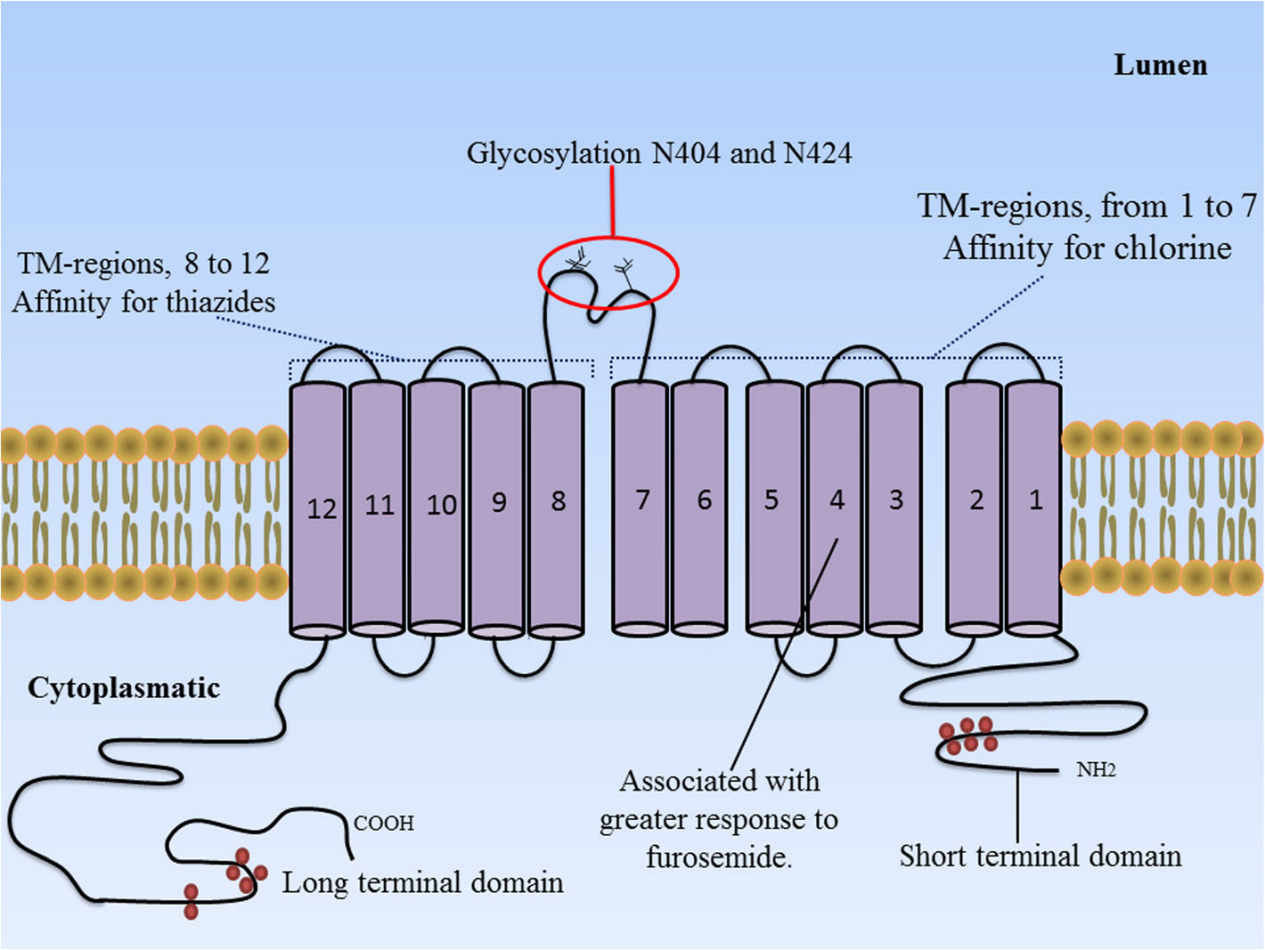
Proposed topology of the thiazide-sensitive Na-Cl co-transporter. The NCC is a protein composed of a central hydrophobic domain containing 12 transmembrane regions (see from right to left) interconnected by six extracellular handles and five cytoplasmic handles. The glycosylation is present on the large extracellular loop between the 7th and 8th membrane-spanning segment, which is essential in trafficking proteins to the cell surface. Also, the central hydrophobic domain is flanked by a short amino-terminal domain (NH_2_) and a long carboxyl-terminal domain (COOH), which are located inside the cell [27, 28]. Figure adapted from Gamba G [29].

**Fig. 2:**
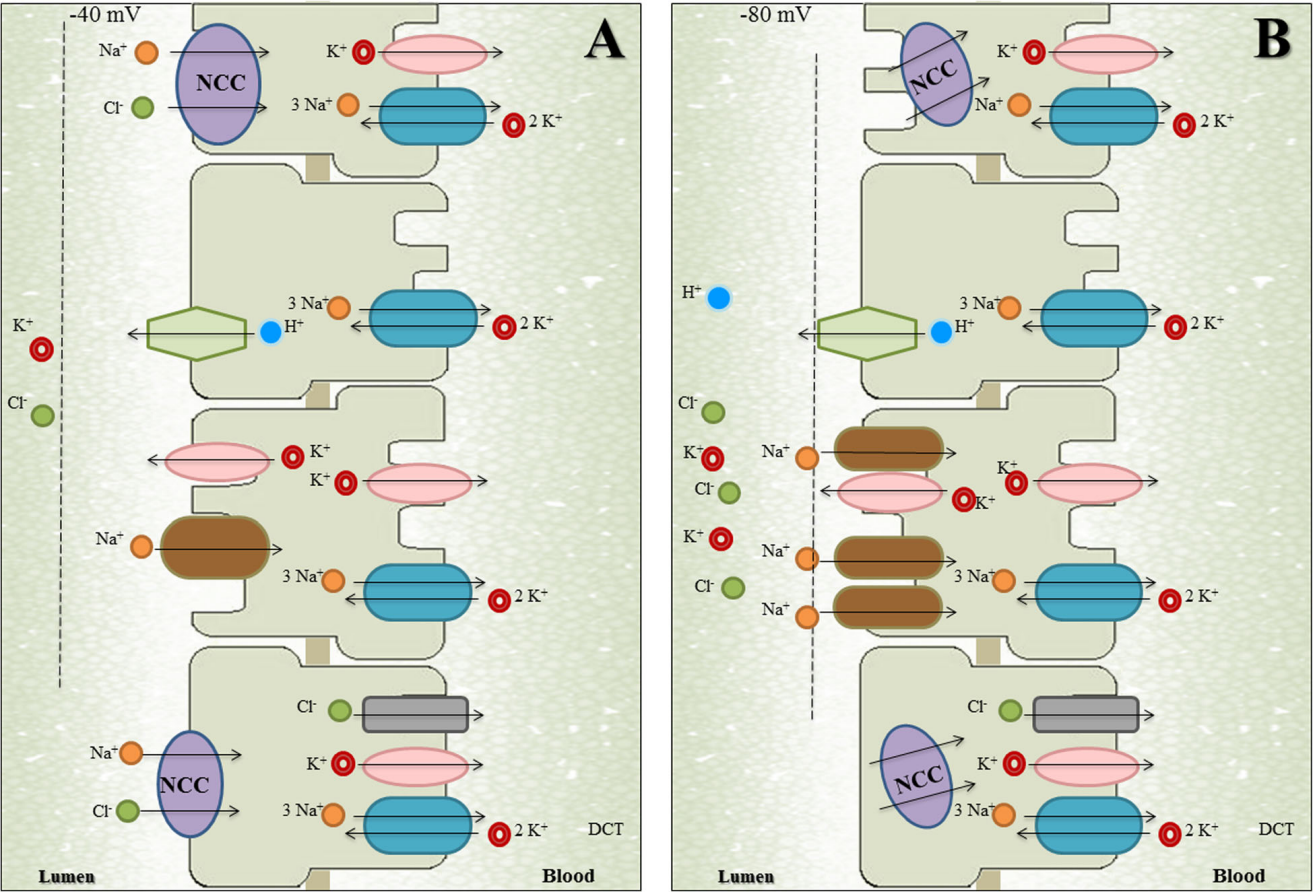
**a** Normal dynamics of reabsorption in the distal nephron. Most of the Na^+^ and Cl^−^ reaching the distal nephron is reabsorbed by NCC in the distal convoluted tubule (DCT) and a smaller percentage is reabsorbed by ENaC in the connecting tubule (CNT) and cortical collecting duct (CCD). However, ENaC reabsorbs exclusively Na^+^ without Cl^−^ in an electrogenic way, which generates a transepithelial potential of − 40 mV. This negative potential in the tubular light favors the secretion of K^+^ and others protons. **b** Abnormal dynamics of reabsorption in the distal nephron. In individuals with Gitelman syndrome or other salt-losing tubulopathies with DCT defects, the dysfunction in NCC (by inactivating mutations in SLC12A3 gene) leads to a greater arrival of Na^+^ and Cl^−^ to CNT/CCD, which favors Na^+^ reabsorption mediated by ENaC. Thus, the increase in the electrogenic reabsorption of Na^+^ increases the transepithelial potential and this produces greater tubular secretion of potassium and others protons (as magnesium and sodium) [35, 36]. **Abbreviations:** CLCNKB: chloride voltage-gated channel Kb; ENaC: epithelial sodium channel; NCC: thiazide-sensitive Na-Cl co-transporter; ROMK: renal outer medullary potassium channel. Figure adapted from Seyberth et al [37]

Diabetic nephropathy (DN) is the most common cause of end-stage renal disease (ESRD) in most of the countries worldwide [1, 2]. The increasing incidence of this condition has become a serious public health problem in terms of both mortality and medical costs [3–5]. Clinical findings of diabetic nephropathy include a decline in estimated glomerular filtration rate (eGFR) [6–8] as well as a progressive increase in urinary albumin excretion [9, 10], associated with an increase in blood pressure and subsequent risk of renal failure [11, 12]. These pathophysiological findings have been related as a consequence of structural abnormalities linked with a rapid renal deterioration, including: *a)* decrease in the number and/or density of podocytes [13, 14], *b)* glomerular basement membrane thickening [15, 16], *c)* progressive expansion of mesangial matrix [17, 18], *d)* tubulointerstitial fibrosis and overt proteinuria [19, 20], eventually a leading cause of glomerulosclerosis and end-stage renal disease [21, 22]. In this context, several epidemiological studies suggest that multiple genetic factors are involved in susceptibility of the pathogenesis of diabetic nephropathy, which has led to extensive research to identify the genes implicated in the development and progression of this condition [16, 23–25]; one of these genes is the solute carrier family 12 member 3 (SLC12A3) gene [16]. The SLC12A3 gene is located on chromosome 16q13 and is specifically expressed in the kidneys, where it encodes a thiazide-sensitive Na–Cl co-transporter (NCC), which is the major salt reabsorption pathway in the distal convoluted tubule (DCT) and located just after the macula densa at the beginning of the aldosterone-sensitive nephron [26, 27]. The NCC is a protein with a molecular weight of 150 kDa with approximately 1002 to 1028 amino acid residues. It is able to form dimers, and it is likely to work as a dimer [26, 27]. The NCC is glycosylated at two sites (N404 and N424) located in the long extracellular loop and it is the site of action of the diuretic thiazide that is frequently administered to patients with T2DM suffering from diabetic nephropathy (see Fig. 1). For a review see Gamba [27]. Furthermore, the inactivation of the Na-Cl co-transporter (SLC12A3) gene is known for being responsible for Gitelman Syndrome (GS), an autosomal recessive renal tubular disorder characterized by hypokalaemia, marked metabolic alkalosis, hypomagnesemia, hypocalciuria, as well as renal potassium and magnesium wasting [30, 31]. Recently, more than 100 different mutations in this gene have been described in patients with GS, in whom a kidney dysfunction has also been linked with abnormalities in the glucose metabolism [30, 32, 33]. Unfortunately, the precise molecular mechanisms linking T2DM and GS are not well understood. However, dysfunction in NCC could be one of the main causes through which insulin resistance in T2DM-individuals leads to chronic hyperglycaemia state and consequently to diabetic nephropathy risk [32, 33]. This assumption is supported by: *(a)* it has been suggested that most of alterations in SLC12A3 gene are inactivating mutations that impair gene transcription or translation in patients with T2DM and GS [33, 34], *(b)* This mutational damage would lead to a truncated/alterated NCC cotransporter polypeptide with a loss function causing impaired reabsorption of sodium chloride, potassium and magnesium in the DCT (see Fig. 2) [38, 39], *(c)* both hypokalaemia as well as hypomagnaesemia have been related to cause insulin secretion abnormalities [33, 40]. Thus, the molecular alterations in this gene could be a key to explain the strong association in both disorders with diabetic nephropathy*.* That is the reason why this systematic review was focused on identifying studies that associated the Arg913Gln variation of SLC12A3 gene with the diabetic nephropathy in T2DM and GS.

### PICOS question

How the Arg913Gln variation of SLC12A3 gene influence in the pathophysiology of diabetic nephropathy in individuals with Type 2 Diabetes Mellitus and Gitelman syndrome?

### Type of studies

Both, case-control and follow-up studies were included for this review.

## Methods

### Literature search strategy

**Fig. 3:**
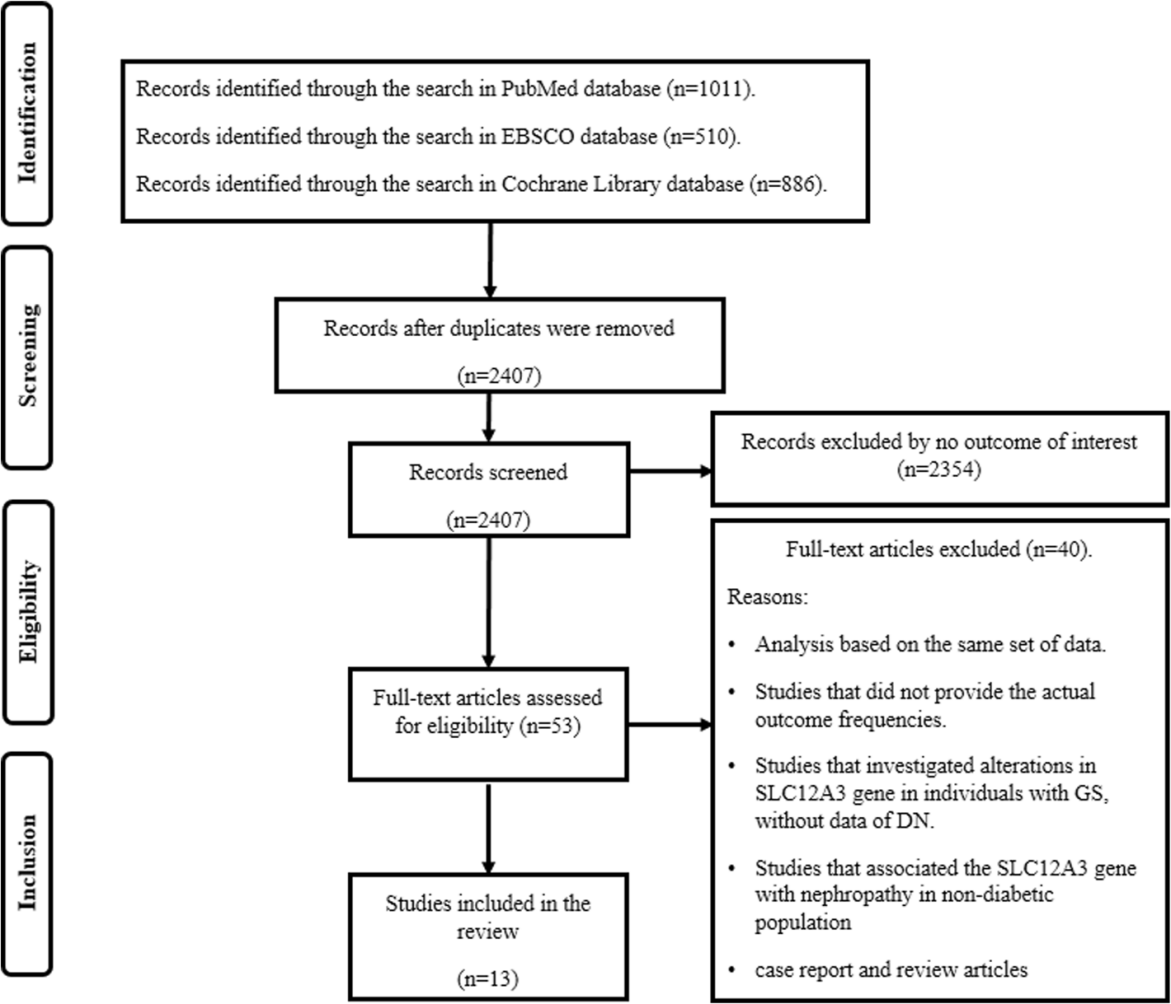
Flow chart showing the search strategy and inclusion/exclusion criteria used in this systematic review

An extensive systematic review of the literature was completed by searching three electronic databases (PubMed, EBSCO and Cochrane Library) from their inception to January 2018 (Additional file 1). From 2407 studies found during the search, only thirteen studies met the inclusion criteria (see Fig. 3). Different combinations of the keywords “SLC12A3 gene AND diabetic nephropathy”, “SLC12A3 gene AND type 2 diabetes mellitus”, “SLC12A3 gene AND Gitelman Syndrome AND diabetic nephropathy”; “SLC12A3 gene AND Gitelman Syndrome AND type 2 diabetes mellitus” were used to screen for potentially relevant studies. The references of all included articles were also screened. The search strategy was conducted in accordance with the PRISMA guidelines (Additional file 2) [41]. The reasons for exclusion as well as the search terms used for PubMed, EBSCO and Cochrane Library databases are displayed in Fig. 3. The quantitative synthesis of the results from the included studies was not possible due to evident methodological heterogeneity, therefore a meta-analysis was not considered.

### Inclusion and exclusion criteria

A study was included in this systematic review only if it met all the following inclusion criteria: *1)* Studies that analyzed the SLC12A3 gene in individuals with T2DM and/or GS. *2)* Use of at least one analysis investigating the association between the Arg913Gln variation of SLC12A3 gene with diabetic nephropathy. *3)* Use of a case–control or follow-up design; *4)* Investigation of type 2 diabetes mellitus in individuals with Gitelman’s syndrome, with a history of diabetic nephropathy and/or end-stage renal disease; 5) Inclusion of reports written only in English language. The analysis based on the same set of data were excluded. The studies that associated the SLC12A3 gene with nephropathy in non-diabetic population as well as studies that investigated alterations in this gene in individuals with Gitelman’s syndrome, without data of diabetic nephropathy were not included. Duplicate publications and case reports, were also excluded. The Fig. 3 shows the study selection process.

### Data extraction

Two investigators (E.D.C. and C.J.G.) extracted the data independently. For conflicting evaluations, an agreement was reached after a discussion. Briefly, for all studies, the following data were extracted from the original publications: first author, year of publication, study design, population analysed, number of patients included (only those that were diagnosed with type 2 diabetes) and control subjects, main characteristics of individuals, as well as main results of association with diabetic nephropathy for type 2 diabetes mellitus and Gitelman Syndrome.

## Descriptive synthesis

### SLC12A3 gene in T2DM-individuals with diabetic nephropathy

Nine case-control studies were found analyzing the SLC12A3 gene in T2DM-individuals (see Table 1). Kim et al [42], Zhao et al [47], Zhang et al [48] and Bodhini et al [46] analyzed Koreans, Chinese and Indians individuals. These studies reported that the minor allele 913Gln in the SLC12A3 gene was significantly associated with end-stage renal disease. Also, these studies concluded that this genetic variant could predict the risk of increase of albuminuria in individuals with T2DM [42, 46–48]. Also, Abu et al [43] and Nishiyama et al [45] analyzed Asian population (specifically Malaysians and Japanese individuals, respectively), in which it was reported that carriers of the Arg913Gln variation were linked with diabetic nephropathy as well as alterations in albumin excretion. Additionally, these studies reported that the minor allele 913Gln in the SLC12A3 gene could have a protective genetic effect against the development and/or progression of diabetic nephropathy for these populations (*p < 0.01*) [43, 45]. Nevertheless, Tanaka et al [23] also analyzed Japanese individuals and found that substitution of Arg913 to Gln in the SLC12A3 gene could reduce the risk of developing diabetic nephropathy [23]. On the other hand, Yadav et al [44] and Ng et al [24] analyzed Indians and American Caucasians populations, respectively. In both studies none polymorphisms in SLC12A3 gene (including the Arg913Gln variation) were associated with diabetic nephropathy [24, 44]. Additionally, Yadav and colleagues found significant differences in genotype and allelic frequency in the SLC12A3 gene between diabetic subjects and controls *(P < 0.03,* 44]*.* The number of cases (sample size) for studies that associated the SLC12A3 gene with diabetic nephropathy in T2DM ranged from 71 to 583.

**Table 1:** Evidence of studies that associated the SLC12A3 gene with diabetic nephropathy in T2DM

Author (year)	Study design	Country	*N* Sample		Characteristics of individuals with diabetic nephropathy	Major diagnosis	Main results of association with diabetic nephropathy
			Cases	Controls			
Tanaka et al [23] (2003)	Case-control	Japanese	94	94	Female *n* = 31; Male *n* = 63; Duration of diabetes ±SD (years) = 18.6 ± 9.7; HbA1c (%) ± SD = 7.7 ± 1.3; S-Cr (mg/dl) ± SD = 1.37 ± 0.83.	DN	In this study Tanaka and colleagues reported that SLC12A3 -Arg913Gln variation might reduce the risk to develop diabetic nephropathy (OR = 2.53; CI 95% = 1.57–4.09; *p* = 0.000087).
Kim et al [42] (2006)	Case-control	Koreans	177	184	Female *n* = 72; Male *n* = 105; Mean age ± SD = 61 ± 9 Duration of diabetes ± SD (years) = 18 ± 8; HbA1c (%) ± SD = 7.2 ± 1.5; S-Cr (mg/dl) ± SD = 7.03 ± 2.4.	DN/ESRD	This study reported that the Arg913Gln variation of SLC12A3 gene is associated with ESRD resulting from diabetic nephropathy in Korean population.(OR = 2.30; CI95% = 1.32–4.00; *p* = 0.003).
Ng et al [24] (2008)	Case-control	American Caucasians	295	174	Female *n* = 116; Male *n* = 179; Duration of diabetes ± SD (years) = 17 ± 8; HbA1c (%) ± SD = 8.0 ± 1.6; S-Cr (mg/dl) ± SD = 7.03 ± 2.4; Cases with CRF/ESRD (%) = 53.2	DN	In this study none of the SNPs showed any significant association with advanced diabetic nephropathy both in terms of allelic or genotypic distributions (OR = 1.213; 95% CI = 0.775–1.897; *p* = 0.397).
Abu et al [43] (2014)	Case-control	Malaysians	124	784	Female *n* = 56; Male *n* = 68; Duration of diabetes ± SD (years) = 13 ± 8; HbA1c (mmol/mol) ± SD = 8.8 ± 2.2; S-Cr (mg/dl) ± SD = 2.23 ± 1.94; GFR ± SD (ml/min/1.73 m2) = 59.8 ± 35.5; ACR ± SD = 269.8 ± 341.2	DN	This study reported that SLC12A3 -Arg913Gln variation was associated with diabetic nephropathy (OR = 0.547; 95% CI = 0.308–0.973; *p* = 0.038) and T2DM (OR = 0.772; 95% CI = 0.612–0.973; *p* = 0.028). In addition, Abu and colleagues indicated that the minor 913Gln allele in this gene could confer a protective effect in the DN.
Yadav et al [44] (2014)	Case-control	Indians	202	197	Female *n* = 62; Male *n* = 140;Duration of diabetes ± SD (years) = 13.81 ± 7.01; HbA1c (mmol/mol) ± SD = 8.0 ± 2.0; S-Cr (mg/dl) ± SD = 1.55 ± 0.97; FBS (mg/dL) ± SD = 139 ± 53	DN	This study reported significant differences in the Arg913Gln variation of SLC12A3 gene between diabetic subjects and controls (*P* < 0.03).
Nishiyama et al [45] (2005)	Case-control	Japanese	71	193	Female *n* = 18; Male *n* = 53; Duration of diabetes ± SD (years) = 8.5 ± 0.9; HbA1c (mmol/mol) ± SD = 8.08 ± 0.13.	DN	In this study it was reported that SLC12A3 -Arg913Gln variation was linked with albumin excretion (OR = 0.09; 95% CI = 0.01–0.92; *p* = 0.043), and that the +78A allele could have a protective effect against the development of DN among this population.
Bodhini et al [46] (2016)	Case-control	Indians	583	601	Female *n* = 207; Male *n* = 376; Duration of diabetes ± SD (years) = 19 ± 8; HbA1c (%) ± SD = 8.6 ± 1.9; FBS (mg/dL) ± SD = 160.3 ± 73.9; S-Cr (mg/dl) ± SD = 1.2 ± 0.89.	DN	In this study it was showed that the individuals carrying of the SLC12A3 -Arg913Gln variation had a significant association with DN (OR = 1.52; 95%CI = 1.06–2.18; *p* = 0.020).
Zhao et al [47] (2009)	Case-control	Chinese	163	96	Clinical and sociodemographic characteristics were not reported because the full article was not found	DN	This study concluded that Arg913Gln polymorphism of SLC12A3 gene may predict the risk of increase of albuminuria in patients with T2DM in Chinese population.
Zhang et al [48] (2017)	Case-control	Chinese	221	151	Female *n* = 84; Male *n* = 137; Duration of diabetes ± SD (years) = 17.7 ± 0.6; HbA1c (%) ± SD = 7.1 ± 0.2; FBS (mg/dL) ± SD = 147.7 ± 5.4.	DN/ESRD	In this study it was suggested that the SLC12A3-Arg913Gln variation is associated with a high risk of DN/ESRD in Chinese T2DM patients undergoing haemodialysis.

Abbreviations: *ACR* albumin-creatinine ratio, *DN* diabetic nephropathy, *ESRD* End-Stage Renal Disease, *FBS* Fasting blood sugar, *S-Cr* Serum creatinine, *SD* Standar Desviation, *SLC12A3 gene* solute carrier family 12 member 3-gene, *T2DM* type 2 diabetes mellitus

### SLC12A3 gene in GS-individuals with diabetic nephropathy

Four studies that analyze the SLC12A3 gene in GS-individuals were found (see Table 2). Ren et al [33], Tseng et al [35] and Yuan et al [32] analyzed an Asian population, in which abnormalities in glucose metabolism (that is, high glycemia levels) and insulin secretion in GS-individuals were reported. Hence, homeostasis model assessment of insulin resistance in these patients was significantly higher (*p* < 0.05 for these studies) [32, 33, 35]. Additionally, Tseng and colleagues indicated that GS-individuals may be at increased risk for the development of T2DM and diabetic nephropathy [35]. Moreover, Balavoine et al [49] analyzed a French population, in which also an increased susceptibility to glucose intolerance was associated with renal failure in GS heterozygous patients. Likewise, Balavoine and colleagues found that GS was more severe in individuals with heterozygous mutant alleles than in those with homozygous mutant alleles in SLC12A3 gene [49]. The number of cases (sample size) for studies that associated the SLC12A3 gene with diabetic nephropathy in GS ranged from 16 to 117.

**Table 2:** Evidence from studies involving the Gitelman’s Syndrome individuals in risk of nephropathy and T2DM

Author (year)	Study design	Population	*N* Sample		Characteristics of GS individuals with risk of nephropathy and T2DM	Major diagnosis	Main results of association in GS individuals with risk of nephropathy and T2DM.
			Cases	Controls			
Yuan et al [32] (2017)	Case-control	Chinese	28	20	AUC glucose (mmol·h/L) ± SD = 17.4 ± 5.1; AUC insulin (μU·h/mL ± SD = 221.5 ± 128.1; ISSI±SD = 81,389 ± 34,680; QUICKI±SD = 0.6 ± 0.1;	GS/DN	This study reported abnormalities in glucose metabolism and insulin secretion in GS patients. It was also observed that the areas under the serum glucose curves were higher in the GS patients than those in the healthy controls (*p* = 0.02).
Tseng et al [35] (2012)	Follow-up	Taiwanese	117	NA	Female *n* = 47; Male *n* = 70; Duration of diabetes ± SD(years) = 23 ± 3; FBS ± SD = 131–225; S-Cr (mg/dl) ± SD = 2.2 ± 1.0	GS/DN	This study reported that a large proportion of GS-patients had triple SLC12A3 mutations. Also, these individuals showed an increased risk for the development of chronic kidney disease and T2DM.
Ren et al [33] (2013)	Case-control	Chinese	16	12	AUC glucose (mEq · h/L) = 16.1(IQR 12.5–25.4); AUC insulin (μU · h/mL) = 81.0 (IQR 58.9–138).	GS/DN	This study found that GS patients showed a higher glucose level compared with control group (p < 0.05).Also, Ren and colleagues observed that GS patients showed a delay of insulin secretion peak which was observed 120 min after a glucose load.
Balavoine et al [49] (2011)	Follow-up	French	15	5	Age (years) ± SD = 35 ± 15; BMI(kg/m2) ± SD = 24.3 ± 6.7; T2DM = 20% (3/15); S Cr (mg/l) ± SD = 8.2 ± 1.1.	GS/DN	In this study was found an increased susceptibility to glucose intolerance in GS heterozygous patients. Additionally, Balavoine and colleagues confirmed the presence of mutations of the SLC12A3 gene in 80% of cases.

Abbreviations: *AUC* area under curve, *CKD* chronic kidney disease, *DN* diabetic nephropathy, *FBS* Fasting blood sugar, *GS* Gitelman’s syndrome, *IQR* interquartile range, *ISSI* insulin secretion-sensitivity index, *NA* not applicable, *SD* standard desviation, *SLC12A3 gene* solute carrier family 12 member 3-gene, *T2DM* type 2 diabetes mellitus, *QUICKI* quantitative insulin sensitivity check index

## Discussion

In this systematic review, the association of the Arg913Gln variation of SLC12A3 gene with diabetic nephropathy in individuals with T2DM and GS was explored. In an important way, it should be noted that a meta-analysis has studied the SLC12A3 gene in association with diabetic nephropathy in T2DM [43]. However, this study only included a small group of studies (four studies) in its review, and did not include GS-individuals with diabetic nephropathy and/or T2DM [43]. In regard to this variation of SLC12A3 gene in T2DM-individuals with diabetic nephropathy, this systematic review found a genetic association in most the studies included [42, 43, 45–48]. However, two of these studies indicated that others genetic variants in this gene could have a protective effect in this disease [43, 45], which also coincides with the findings by Tanaka *et al* [23], who indicated that SLC12A3 -Arg913Gln gene variation could reduce the risk to develop diabetic nephropathy in T2DM [23]. A possible reason for this discrepancy in these results may be related to a wide variety of genetic factors are involved in diabetic nephropathy in a complex form, in which these variants in the SLC12A3 gene may be present in regulating regions, such as promoter, intron sequences or 5′ and 3′ non-coding regions, and the effect of such polymorphisms may not have been detected in the relatively small populations of T2DM-individuals with diabetic nephropathy. Regarding the SLC12A3 gene in GS-individuals with diabetic nephropathy, when the results of these studies were analyzed globally [32, 33, 35, 49], it was observed an increased susceptibility to glucose intolerance as well as abnormalities in insulin secretion, which were associated with renal failure within this group of patients. The assumption that Gitelman syndrome is caused by an alteration in the thiazide-sensitive sodium-chloride co-transporter in the distal convoluted tubule has recently been proven by the identification of several mutations (mainly amino acid substitutions) in the SLC12A3 gene, where also a large number of deep intronic mutations could be linked with the development and/or progression of diabetic nephropathy [32, 35]. Therefore, it is likely that the majority of these alterations are indeed harmful mutations and not innocuous polymorphisms linked with kidney disease. Likewise, phenotypic variability in terms of biochemical alterations (such as hypomagnesemia and hypokalemia), could also play an important role in the pathogenesis of DN in GS-individuals, in which this heterogeneity has even been described in case reports within GS families with identical genetic mutations [34, 50]. Unfortunately, the biochemical mechanisms by which magnesium and potassium deficiency induces or worsens T2DM in GS-individuals are not well understood, but it is thought that these electrolytic alterations may induce altered cellular glucose transport, defective post receptor insulin signaling, impaired secretion of biologically active insulin as well as altered insulin-insulin receptor interactions [51, 52]. Also, it is interesting that the induction of magnesium and potassium deficiency has been shown to reduce insulin sensitivity in subjects without T2DM, whereas that supplementation of these electrolytes during relatively long periods has been shown to improve glucose handling in elderly subjects without T2DM [53, 54]. Additionally, it has been indicated that complications of T2DM (e.g. diabetic nephropathy, retinopathy, neuropathy, and foot ulcerations) are more severe in the presence of chronic hypomagnesaemia of any cause [51]. The present study also has some limitations inherent to the studies included. For instance, only studies published in journals were included in this review, since studies with negative results are often not published, resulting to an overestimation of genetic implications. Also, the design and the small number of studies included in this systematic review limit the ability to make causal inferences; since the studies included in this review were not clustered in terms of ethnicity. Hence, failure to account for ethnicity-specific interactions between genetic polymorphisms and environmental factors could also contribute to the pattern of results observed in individuals with diabetic nephropathy and/or end-stage renal disease. Nevertheless, the potential limitation of the present study is the substantial methodological heterogeneity of the findings obtained in this systematic review.

## Conclusions

The present systematic review provides evidence to support that the Arg913Gln variation of SLC12A3 gene is associated with the diabetic nephropathy in individuals with T2DM and GS. Because most of the individuals included in this research belonged to Asian populations, the findings of this systematic review need to be confirmed and replicated in other ethnicities worldwide with heterozygous carriers. Moreover, the recommendation for future GWAS as strategy for unravelling genetic complexity on diabetic nephropathy outcomes in individuals with T2DM and GS. Thus, the findings in these studies could be supportive in replicating existing evidence and in revealing genuine genetic effects to confirm the role of the polymorphisms of candidate genes linked with end-stage renal disease with an observable trait.

## Supplementary information

**Additional file 1.** Search strategy terms and results.

**Additional file 2.** PRISMA Checklist.

## Data Availability

All data generated or analysed during this systematic review are included in the published article. (Additional file 1 & Additional file 2).

## Abbreviations

CCD: Cortical collecting duct
CLCNKB: Chloride voltage-gated channel Kb
CNT: Connecting tubule
DCT: Distal convoluted tubule
DN: Diabetic nephropathy
eGFR: estimated glomerular filtration rate
ENaC: Epithelial sodium channel
ESRD: End-stage renal disease
GS: Gitelman’s syndrome
GWAS: Genome-wide association studies
NCC: thiazide sensitive Na-Cl co-transporter
ROMK: Renal outer medullary potassium channel
SLC12A3: Solute carrier family 12 member 3
T2DM: Type 2 diabetes mellitus
