# Supplementary material for: Arg913Gln variation of SLC12A3 gene is associated with diabetic nephropathy in type 2 diabetes and Gitelman syndrome: a systematic review
Source: BMC Nephrol. 2019 Oct 28;20:393. doi: 10.1186/s12882-019-1590-9 (PMC6819471; doi:10.1186/s12882-019-1590-9)
Supplement: Supplementary file 1 — Additional file 1. Search strategy terms and results. [file 12882_2019_1590_MOESM1_ESM.docx]

**Search strategy terms and results**

| PUBMED, searched from January 1, 1985 to January 31, 2018, case–control and/or follow-up studies in humans | | |
| --- | --- | --- |
| 1 | SLC12A3 gene AND diabetic nephropathy | 4265 |
| 2 | SLC12A3 gene AND type 2 diabetes mellitus | 5223 |
| 3 | SLC12A3 gene AND Gitelman Syndrome AND diabetic nephropathy | 5412 |
| 4 | Combine #1 AND # 2 AND # 3 | 7966 |
| 5 | Filters: English | 1011 |

| EBSCO, searched from January 1, 1985 to January 31, 2018, case–control and/or follow-up studies in humans | | |
| --- | --- | --- |
| 1 | SLC12A3 gene AND diabetic nephropathy | 4469 |
| 2 | SLC12A3 gene AND type 2 diabetes mellitus | 5823 |
| 3 | SLC12A3 gene AND Gitelman Syndrome AND diabetic nephropathy | 5328 |
| 4 | Combine #1 AND # 2 AND # 3 | 6956 |
| 5 | Limit to: English | 510 |

| Cochrane, searched from January 1, 1985 to January 31, 2018, case–control and/or follow-up studies in humans | | |
| --- | --- | --- |
| 1 | SLC12A3 gene AND diabetic nephropathy | 5812 |
| 2 | SLC12A3 gene AND type 2 diabetes mellitus | 8951 |
| 3 | SLC12A3 gene AND Gitelman Syndrome AND diabetic nephropathy | 3986 |
| 4 | Combine #1 AND # 2 AND # 3 | 6954 |
| 5 | Filters: English | 886 |
